# Surgical Treatment of Empyema of the Thorax

**Published:** 1919

**Authors:** 


**Surgical Treatment of Empyema of the Thorax.** By HOWARD LILIENTHAL, Lieut.-Col. M. C.

Lilienthal advises early treatment of thoracic empyemas, and classifies them surgically into the three following groups :

A. Acute, with the chest full of pus and immediate danger to life — cyanosis, dyspnea, sepsis present.

B. Sub-acute and chronic, often long unrecognized. Little fever; cyanosis absent; shortness of breath on exertion, but otherwise no dyspnea.

C. Chronic cases with thoracic sinuses and undrained cavities, following operation.

He advises the following procedures in the different groups :

*Group A.* 1. X-ray if there is time.

2. Minor thoracotomy, see below.

3. If there is not what may be called "normal" improvement within six days following minor thoracotomy, fluoroscopy and picture in the erect posture. If collections of fluid are seen, perform major thoracotomy as described below.

*Group B.* 1. X-ray and fluoroscopy in the erect posture. In these cases the affected side is contracted and the ribs closer together than on the sound side, whether the empyema is sacculated or general. Stereoscopic study is desirable.

2. Major thoracotomy, see below, unless the empyema is encapsulated and occupies only a small part of the chest, when it is desirable to resect one or two ribs with the periosteum, directly over the empyema, packing the cavity lightly with gauze and treating it as an abscess. The healthy neighboring pleura should not be entered, and lung mobilization is usually unnecessary.

*Group C.* 1. Careful X-ray study with fluoroscopy and pictures. Cases should be selected for operation on considering the number of months after primary operation, the character and shape of the cavity and the power of the patient to fill this cavity by distending the lung by blowing or straining.

2. Preparation for operation by disinfection for four to six days by Carrel's method. (See paragraph 1, under Thoracoplasty with Lung Mobilization [Lilienthal].)

3. Thoracoplasty with lung mobilization. (Described below.) No collapsing operations of the chest wall.

Lilienthal then describes the operations which he has devised or modified.

*Minor Intercostal Thoracotomy.* Posture on the well side slightly toward the abdomen; knees and hips flexed; pillow between the knees and another between the thorax; legs held by straps passed around the table; thorax kept in position by sand bags. Arm behind patient as in Sims' position.

Anesthesia, local.

A short incision close to the upper border of the rib in the 7th or 8th intercostal space, posterior axillary line, with insertion of a small thick-walled tube, connected with a long tube which passes below the surface of an antiseptic solution in a bottle beside the bed. Dressings held in place by adhesive plaster; no bandage.

*Major Thoracotomy.* Anesthesia, general, preferably by the intrapharyngeal method. (See below.)

Posture: patient on the well side slightly toward the abdomen; knees and hips flexed; pillow between the knees; legs held by straps passed around the table; thorax kept in position by sand bags; a cushion under patient so as to enlarge the intercostal spaces on the diseased side; arm behind patient as in Sims' position.

Incision in 7th interspace hugging the upper border of the rib, from its angle to the cartilage. The vessels in the latissimus dorsi, serratus magnus and other muscles are caught before they are cut. Pleura is entered by short incision and fluid allowed to escape slowly, or, better, withdrawn by suction apparatus. Pleural opening enlarged with scalpel or scissors until entire length of the wound has entered chest. Look out for diaphragm anteriorly. Insert blades of powerful rib retractor and spread them gradually. A separation of from four to six inches is usually possible. Remove all fluid and lymph coagula. Hemorrhage is insignificant up to this point, and in empyema there will be no respiratory embarrassment. Explore by sight and touch all parts of the visible thoracic cavity. Carefully break down any soft adhesions to the chest wall, mediastinum, and diaphragm, and separate the pulmonary lobes. If, however, as is usual, a more or less confining membrane has already bound down the lung so that all landmarks are obliterated, make an incision with the scalpel through this membrane from that part of the cavity nearest the apex to the base. Usually this incision will be immediately spread by the underlying lung which will force its way toward the chest wall. Lateral incisions at right angles to the first may now be made with scalpel or scissors when still freer lung expansion will be secured. This procedure also is rarely accompanied by hemorrhage.

A slight wound of the lung with the appearance of fine bubbles need not cause alarm; coarse bubbling, however, would signify entrance into a bronchus. In the recent cases this may always be avoided. It is not necessary, except in the presence of secondary pus collections between the lung and the costal pleura, to break down all adhesions to the chest wall. It is most important, however, to lift the lower lobe from the diaphragm, for here separate pus collections will not infrequently be found, especially when the lower lobe appears atelectatic. Care must be taken not to confuse this lobe with the diaphragm itself. When full mobilization has occurred, the lung will crowd toward the chest wall and into the wound on insufflation by the anesthetist, or upon coughing or straining by the patient. The ribspreader may now be removed, and the wound closed by layer suture; the muscles with chromicized catgut No. 2, the skin with silk sutures. It is not necessary to draw the ribs together by special suture. The separation of an inch and a half or more between the ribs will return to the normal about ten days following the operation. Openings for drainage may be left at the angles of the wound where splint tubes or rubber dam may be inserted.

This large wound rarely becomes infected, probably because there is free drainage into the pleural cavity, the skin healing per primam. The usual dry gauze dressing is now applied and held in place by adhesive straps which must not encircle the chest.

Lilienthal describes his operation thoracoplasty with lung mobilization, as given below :

Preliminary disinfection by Carrel Dakin method. Great care is necessary in the intrathoracic application of Dakin's fluid because even a minute and unsuspected bronchial fistula will lead to the actual "gassing" of the patient.

Anesthesia, intrapharyngeal insufflation. (See below.)

Posture : as in major thoracotomy.

Incision : in 6th and 7th interspace as in major thoracotomy. The chest in these cases, however, will be found rigid and separation of the ribs impossible. Therefore, from one to three, or even four ribs, will have to be divided with bone forceps at the posterior angle of the wound, upward or downward or both as the case may be. The rib spreader may then be inserted and the entire cavity laid open to the most searching exploration. Try to avoid making the incision through the old sinus. Lung mobilization is then practiced by incising the visceral layer of the confining membrane from the apex to the base of the cavity. In these cases complete exploration of the uninvolved thorax is unnecessary, because the X-ray will have demonstrated the location and general form of the cavity. If the membrane cannot readily be peeled off, in spite of the incision at right angles to the first, it will be necessary to make numerous crosshatchings (Ranschoff) over the entire visceral wall of the cavity. The anesthetist will then insufflate and distend the lung as far as possible. If complete distension has been secured the prognosis is excellent. Very rarely one encounters fibrosis of the lung so that expansion will not take place by insufflation, coughing or straining. In these rare cases it is probable that a collapse operation, at some future time may be avoidable. Having mobilized the lung a forceps is passed into the cavity from the old sinus, and a piece of iodized gauze is drawn through from the wound of the chest outward, reaming out the tract. A piece of rubber tubing is then drawn through the old wound just long enough to reach the inside of the chest. If there is a tendency for the divided ends of the ribs to overlap, a short piece may be resected so that the ends do not touch. The wound is now completely closed by layer suture, chromicized catgut being employed for the muscles, and silk for the skin.

The author considers the best " rib spreader " is that devised by himself. It has been made in the A. E. F. by Sergeant Herrling, under the direction of Second Lieutenant Frank B. Donovan, A. S. S. C., Co. 8, First Motor Mechanics' Regiment.

The after-treatment devised by Lilienthal is as follows : morphine in full doses should be given for the first twelve to twenty-four hours. The patient is supposed to keep the lung distended by blowing exercises as soon as he is well out of his anesthesia. In the war hospitals a simple blowing apparatus can be made by attaching a rubber tube to a pneumatic pillow, and tying a string around it so as to cause a stricture through which air is blown with difficulty. A profuse discharge necessitating frequent changes of external dressings will be present for the first two or three days. Intrathoracic conditions during convalescence must be observed by means of the X-ray, preferably by fluoroscopy. There should be complete healing within four weeks in simple major thoracotomy, and it has been known to occur in ten days. The operation is rarely accompanied by severe shock, in spite of its apparent magnitude.

In discussing anesthesia in lung surgery the author says that the administration of ether by intrapharyngeal insufflation is a simple matter. The apparatus required is a foot bellows or hand bulb with secondary bulb like that of Paquelin's cautery, and an ether bottle with two tubes through the stopper arranged so that the ether vapor passes through a tube or catheter. The patient is first anesthesized in the usual manner, then this tube or catheter is passed into one nostril not farther than the pharynx, or a distance of say 3 1/2 inches, which should be clearly marked on the tube with a piece of adhesive plaster so that the proper length may not be exceeded. Gentle blowing will drive the ether vapor into the pharynx where there is maintained an increased atmospheric pressure. Increasing the force of the stream of vapor will slow the respiration of the patient. If the ether is to be intermitted, air may be forced through this same tube. To distend the lung it is necessary to close the patient's opposite nostril and place the hand over his closed mouth. The operator will be able to guide the anesthetist as to the proper amount of force to be used in pumping. If the tube is too long there is danger that it may pass into the deeper pharynx or even into the esophagus, causing ether distension of the stomach. This, however, should never occur.

If nitrous oxide is the anesthetic employed, no bellows will be required.

The author considers it important that no liquid ether can possibly be blown into the patient's pharynx.

